# Vital Signs: Fruit and Vegetable Intake Among Children — United States, 2003–2010

**Published:** 2014-08-08

**Authors:** Sonia A. Kim, Latetia V. Moore, Deborah Galuska, Ashton P. Wright, Diane Harris, Laurence M. Grummer-Strawn, Caitlin L. Merlo, Allison J. Nihiser, Donna G. Rhodes

**Affiliations:** 1 Division of Nutrition, Physical Activity, and Obesity, National Center for Chronic Disease Prevention and Health Promotion, CDC; 2 Division of Population Health, National Center for Chronic Disease Prevention and Health Promotion, CDC; 3 Food Surveys Research Group, Agricultural Research Service, US Department of Agriculture

## Abstract

**Background:**

Eating more fruits and vegetables adds underconsumed nutrients to diets, reduces the risks for leading causes of illness and death, and helps manage body weight. This report describes trends in the contributions of fruits and vegetables to the diets of children aged 2–18 years.

**Methods:**

CDC analyzed 1 day of 24-hour dietary recalls from the National Health and Nutrition Examination Surveys from 2003 to 2010 to estimate trends in children’s fruit and vegetable intake in cup-equivalents per 1,000 calories (CEPC) and trends by sex, age, race/ethnicity, family income to poverty ratio, and obesity status. Total fruit includes whole fruit (all fruit excluding juice) and fruit juice (from 100% juice, foods, and other beverages). Total vegetables include those encouraged in the *Dietary Guidelines for Americans, 2010* (i.e., dark green, orange, and red vegetables and legumes), white potatoes, and all other vegetables.

**Results:**

Total fruit intake among children increased from 0.55 CEPC in 2003–2004 to 0.62 in 2009–2010 because of significant increases in whole fruit intake (0.24 to 0.40 CEPC). Over this period, fruit juice intake significantly decreased (0.31 to 0.22 CEPC). Total vegetable intake did not change (0.54 to 0.53 CEPC). No socio-demographic group met the *Healthy People 2020* target of 1.1 CEPC vegetables, and only children aged 2–5 years met the target of 0.9 CEPC fruits.

**Conclusions:**

Children’s total fruit intake increased because of increases in whole fruit consumption, but total vegetable intake remained unchanged.

**Implications for Public Health Practice:**

Increased attention to the policies and food environments in multiple settings, including schools, early care and education, and homes might help continue the progress in fruit intake and improve vegetable intake.

## Introduction

*Dietary Guidelines for Americans, 2010*, recommends that Americans aged ≥2 years eat more fruits and vegetables to add important nutrients that are underconsumed, reduce the risk of heart disease, stroke, and some cancers, and help manage weight (1). Most U.S. residents, including children, consume too few fruits and vegetables. In 2007–2010, 60% of children aged 1–18 years did not meet U.S. Department of Agriculture Food Patterns fruit intake recommendations, and 93% did not meet vegetable recommendations (2). Because of the benefits of eating fruits and vegetables and because childhood dietary patterns are associated with food patterns later in life (3), encouraging children to eat more fruits and vegetables is a public health priority.

*Healthy People 2020* (HP2020) objectives NWS-14 and NWS-15 call for increases in the contribution of fruits to U.S. residents’ diets from 0.5 cup-equivalents per 1,000 calories (CEPC) in 2001–2004 to a 2020 target of 0.9 CEPC and of vegetables from 0.8 CEPC to a 2020 target of 1.1 CEPC.* One cup-equivalent is approximately one small apple, 1 cup of applesauce, 1 cup of 100% juice, or 12 baby carrots.† The Dietary Guidelines emphasizes that the majority of fruit consumed be whole fruit, rather than juice; when juice is consumed, it should be 100% juice (1). The Dietary Guidelines also recommends eating a variety of vegetables, especially dark green, orange, and red vegetables (e.g., broccoli and spinach, carrots and pumpkin, and tomatoes and red peppers, respectively) and legumes.

In spite of some recent evidence of increases in children’s fruit intake (4), trends by fruit and vegetable subgroups have not been reported. This report describes trends in the contribution of fruits and vegetables to children’s diets from 2003–2004 to 2009–2010, overall and by demographic characteristics, using 1 day of 24-hour dietary recall data from the What We Eat in America component of the National Health and Nutrition Examination Survey (NHANES).

## Methods

NHANES is a nationally representative, multistage survey of the noninstitutionalized U.S. civilian population.§ CDC analyzed data for children aged 2–18 years to include preschool and school-aged populations. Consistent with HP2020 methods, CDC used data for children starting at age 2 years. Across the four NHANES survey cycles (2003–2004, 2005–2006, 2007–2008, and 2009–2010) the response rates for persons aged 1–19 years were 81%–90%. A total of 14,865 children aged 2–18 years participated in these four cycles. Trained interviewers collected 24-hour dietary recalls using the U.S. Department of Agriculture (USDA) automated multiple-pass method¶ by proxy for those aged 1–5 years, with proxy assistance for those aged 6–11 years, and directly from participants aged ≥12 years. After excluding those with incomplete data, the final analytic sample was 12,459 participants. All reported single and multi-ingredient foods and beverages were separated into their components and assigned cup-equivalents of fruits and vegetables according to standard recipes using the USDA’s MyPyramid and Food Patterns Equivalents databases corresponding with each NHANES survey cycle.** Total fruit included whole fruit (all forms of fruit, excluding juice) and fruit juice (100% fruit juice plus the 100% fruit juice component of foods and other beverages). Total vegetables included those which the Dietary Guidelines encourages persons to consume (dark green, orange, and red vegetables and legumes), white potatoes, and all other vegetables. Intakes in CEPC of total fruit, total vegetables, and each subgroup were estimated by summing the cup-equivalents consumed from each food and beverage, dividing by caloric intake, and multiplying by 1,000. Mean intake for each survey cycle was age-standardized to the 2000 U.S. population and calculated overall and by sex, age group (2–5, 6–11, and 12–18 years), race/ethnicity (Mexican American, non-Hispanic black, or non-Hispanic white), family income to poverty ratio (<130%, 130% to <349%, and ≥349%), and obesity status (age-specific and sex-specific body mass index ≥95th percentile using the 2000 CDC growth charts††). For family income to poverty ratio, poverty was defined according to federal poverty guidelines.§§ To examine trends in fruit and vegetable intake, average annual change in CEPC per year was calculated using linear regression and was reported as a percent by dividing the annual change by mean intake in 2003–2004. T-tests were used to examine differences in fruit and vegetable subgroups by socio-demographic characteristics in 2009–2010. A p-value of <0.05 was considered statistically significant.

## Results

**FIGURE 1:**
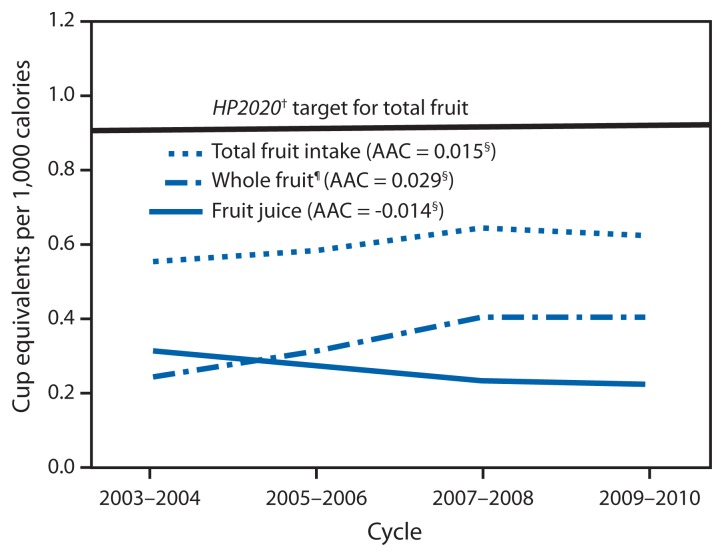
Mean daily intake of fruit in cup-equivalents per 1,000 calories among children aged 2–18 years — National Health and Nutrition Examination Survey, United States, 2003 to 2010* **Abbreviations:** AAC = average annual change; HP2020 = *Healthy People 2020*. * Estimates age adjusted to the 2000 U.S. population. ^†^ Additional information available at http://www.healthypeople.gov/2020/topicsobjectives2020/objectiveslist.aspx?topicId=29. ^§^ Average annual change from 2003 to 2010 calculated using linear regression statistically different from zero at alpha = 0.05. ^¶^ Whole fruit includes all forms of fruit, excluding juice.

Total fruit intake among children significantly increased 0.015 CEPC or 3% of the 2003–2004 baseline amount, per year (0.55 CEPC in 2003–2004 to 0.62 in 2009–2010) (Figure 1). Whole fruit intake significantly increased 0.029 CEPC or 12% per year (0.24 to 0.40 CEPC); fruit juice intake significantly decreased 0.014 CEPC or 5% per year (0.31 to 0.22 CEPC). Total fruit intake increased significantly among males, children aged 6–11 years, children from families with incomes in the 130% to <349% poverty threshold, and obese children (Table 1). Whole fruit intake increased significantly among all socio-demographic groups.

**FIGURE 2:**
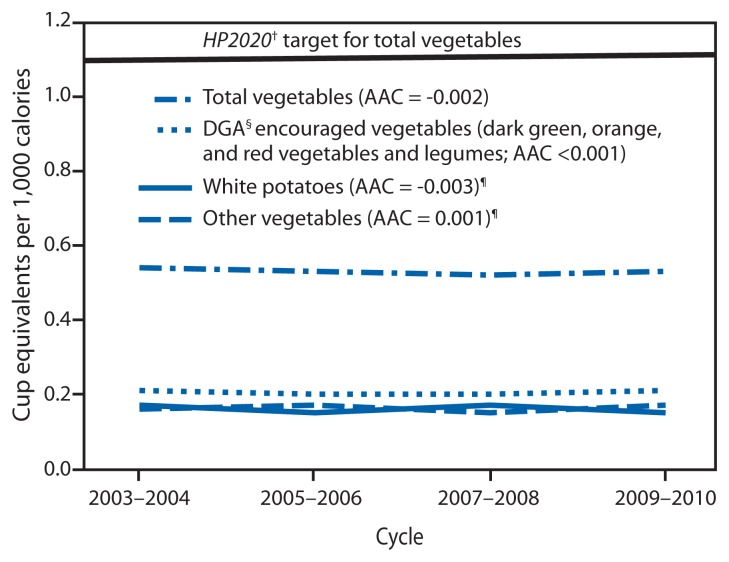
Mean daily intake of vegetables in cup-equivalents per 1,000 calories among children aged 2–18 years — National Health and Nutrition Examination Survey, United States, 2003 to 2010* **Abbreviations:** AAC = average annual change; DGA = Dietary Guidelines for Americans, 2010; HP2020 = *Healthy People 2020*. * Estimates age adjusted to the 2000 U.S. population. ^†^ Additional information available at http://www.healthypeople.gov/2020/topicsobjectives2020/objectiveslist.aspx?topicId=29. ^§^ Available at http://www.health.gov/dietaryguidelines. ^¶^ Estimates for white potatoes and other vegetables overlap across the study period (0.15–0.17 cup-equivalents per 1,000 calories).

Total vegetable and vegetable subgroup intake in CEPC did not change over time (Figure 2). White potatoes accounted for an average of 30% of total vegetable intake over the study period (0.15–0.17 CEPC) (Figure 2) and were consumed mainly as less healthy forms of potatoes (e.g., fried potatoes and potato chips) (0.09–0.11 CEPC over the study period). Trends in total vegetable intake in CEPC were similar across socio-demographic groups, except for slight but significant decreases among Mexican Americans (driven by a significant decrease in Dietary Guidelines-encouraged vegetables) and non-Hispanic black children (driven by a significant decrease in other vegetables) (Table 2).

Disparities in total fruit intake existed by age in 2009–2010. Children aged 2–5 years consumed significantly more fruit in CEPC than older children (p<0.01 for differences) (Table 1). Females consumed more total vegetables in CEPC than males, and children aged 12–18 years consumed more vegetables in CEPC than younger children (p<0.05 for differences). Mexican American children consumed more vegetables in CEPC than non-Hispanic black children (p=0.01). No socio-demographic group met the HP2020 total vegetable target and only children aged 2–5 years met the total fruit target.

### Discussion

From 2003–2004 to 2009–2010, children’s total fruit intake per 1,000 calories increased an average of 3% per year, or a total of 13% between the two periods. Whole fruit increased an average of 12% per year, or 67% over the period, while fruit juice decreased an average of 5% per year, or 29% over the same period. Total vegetable intake per 1,000 calories remained unchanged. Children aged 2–5 years consumed 0.51 CEPC (about one half of a small apple for every 1,000 calories eaten) more total fruit than children aged 12–18 years. No socio-demographic group met the HP2020 total vegetable target and only children aged 2–5 years met the total fruit target. Increases in whole fruit intake and decreases in fruit juice intake are both encouraging patterns. The Dietary Guidelines and the American Academy of Pediatrics emphasize that most fruit should be consumed as whole fruit, rather than juice (1,5). Although 100% juice can be part of a healthy diet, it might be easy to over consume (5), and it lacks the fiber of whole fruit (1,5).

Children’s fruit and vegetable consumption might be influenced by taste preferences, repeated exposures to fruits and vegetables, social experiences, and availability (6). Although specific reasons for the increase in fruit intake among children are unknown, a number of policies and programs implemented over the last several years might have contributed. For example, the addition of a voucher for fruits and vegetables worth $6–$10 to the Special Supplemental Nutrition Program for Women, Infants, and Children (WIC) in 2009 might have contributed to increased intake in the last survey cycle (7,8). The federal Fresh Fruit and Vegetable Program, which provides free fruits and vegetables to eligible elementary schools, expanded from a few states in 2002 to all 50 states in 2008 (9). The program increased fruit and vegetable consumption among program participants by about one third of a cup per day, mainly as fruit (9). The Child Nutrition and WIC Reauthorization Act of 2004 required school districts to adopt school wellness policies that included goals for nutrition standards and nutrition education by 2006.¶¶ Multiple states also adopted various policies to improve the food environment within early care and education settings and schools, which might have resulted in increased healthy offerings in these settings (10–12). Although all of those policies and programs encouraged higher intake of fruits and vegetables, vegetable intake did not increase. Evidence suggests that children have a stronger preference for fruits than vegetables, and that it might be easier to increase consumption of fruits than vegetables (6).

Continued efforts are needed to increase children’s fruit and vegetable consumption. Expert bodies have identified parents, schools, early care and education providers, community and business leaders, and state and local officials as stakeholders who might affect the nutrition environments of children. (1,13,14) Among these, schools and early care and education are important settings (13,14) because approximately 60 million children (15,16) are exposed to the food and education provided in these settings. In addition, two recent studies showed that implementing policies about foods offered in schools improved children’s fruit and vegetable consumption (17,18). Furthermore, federal policies and programs can be used to encourage fruit and vegetable consumption in these settings. For example, the Healthy Hunger-Free Kids Act, 2010*** increased the amount and variety of fruits and vegetables served in the National School Lunch and School Breakfast Programs,††† required USDA to establish nutrition standards for all foods sold during the school day, and required that foods offered in early care and education settings through the Child and Adult Care Food Program align with the Dietary Guidelines.§§§ CDC funds state health departments to improve healthy eating at schools and early care and education settings through the State Public Health Actions to Prevent and Control Diabetes, Heart Disease, Obesity, and Associated Risk Factors and Promote School Health.¶¶¶ At least two Let’s Move!**** initiatives support the improvement of children’s dietary quality, including *Let’s Move! Child Care* and *Let’s Move! Salad Bars to Schools*.

**Key Points**

- Eating fruits and vegetables adds important nutrients, helps control weight, and reduces the risks for many serious illnesses.
- Whole fruit intake among children aged 2–18 years increased by 12% of the 2003–2004 baseline amount per year from 2003 to 2010. Fruit juice intake significantly decreased.
- Vegetable intake among children did not change from 2003 to 2010.
- Most children still consume too few fruits and vegetables, in spite of progress. About 60% of children consume fewer fruits than recommended, and 93% of children consume fewer vegetables than recommended.
- Schools and early care and education providers can help continue progress on fruit intake and improve vegetable intake by: 1) meeting or exceeding current nutrition standards for meals and snacks, 2) serving fruits and vegetables whenever food is offered, 3) training staff members to make fruits and vegetables more appealing and ready to eat, and 4) providing nutrition education and hands-on learning opportunities such as growing and preparing fruits and vegetables.
- Additional information is available at http://www.cdc.gov/vitalsigns.

School districts, schools, and early care and education providers can help increase children’s fruit and vegetable consumption by implementing nutrition standards that meet or exceed federal regulations for meals and snacks (19–22). They can bolster these nutrition standards in a number of ways. For example, schools, school districts, and early care and education providers can make fruits and vegetables available whenever food is offered (19), increase the visibility and appeal of fruits and vegetables in cafeterias (19), and ensure that staff members are trained to implement nutrition standards and model healthy behaviors (19,20). They can also provide nutrition education as a part of classroom activities (13,19–21) and within comprehensive health education (19) and offer hands-on learning opportunities (13,19,21,22) that might include food preparation, gardening, and farm-to-school and pre-school programs (19).

The findings in this report are subject to at least five limitations. First, the 24-hour dietary recalls are reported by either parents or children and are subject to recall and social-desirability biases (23,24). Second, estimating fruit and vegetable intake relies on the MyPyramid and Food Patterns Equivalents databases, which disaggregate foods and beverages into cup-equivalents according to standard recipes. Incongruence between recipes and actual foods consumed might introduce measurement error. Trends were estimated from four NHANES cycles from 2003–2004 to 2009–2010. The most recent data available were for 2009–2010. Earlier data were not used because total fruit was not disaggregated into whole fruit and fruit juice in the USDA databases before 2003–2004. Finally, the response rates in NHANES were 81%–90% for persons aged 1–19 years across the study years; lower response rates can result in nonresponse bias. However, NHANES data are weighted to adjust for nonresponse to minimize bias.

Children’s total fruit intake per 1,000 calories increased as a result of increases in whole fruit, but remained well short of national goals. Total vegetable intake per 1,000 calories remains low and unchanged, and about one third of vegetable intake was white potatoes, mainly eaten fried or as potato chips. Increased attention to the policies and food environments where children live, learn, and play, as well as increased opportunities for children to learn about fruits and vegetables, might help continue progress on fruit intake and improve vegetable intake.

## Figures and Tables

**TABLE 1:** Mean intake of total fruit in cup-equivalents per 1,000 calories among children aged 2–18 years, by sex, age, race/ethnicity, family income to poverty ratio, and obesity status — National Health and Nutrition Examination Survey, United States, 2003 to 2010*

	2003–2004			2009–2010				
				
Characteristic	No. surveyed	Mean intake	(95% CI)	No. surveyed	Mean intake	(95% CI)	p-value†	Annual change§
**All children**	**3,348**	**0.55**	**(0.49–0.61)**	**2,830**	**0.62**	**(0.56–0.68)**	**0.032**	**0.015**
**Sex**								
Male	1,663	0.52	(0.44–0.60)	1,475	0.60	(0.53–0.68)	0.021	0.019
Female	1,685	0.59	(0.52–0.66)	1,355	0.64	(0.58–0.71)	0.249	0.010
**Age group (yrs)**								
2–5	706	0.89	(0.76–1.01)	774	0.97	(0.86–1.07)	0.203	0.016
6–11	861	0.51	(0.42–0.60)	1,058	0.61	(0.55–0.67)	0.015	0.020
12–18	1,781	0.43	(0.36–0.50)	998	0.46	(0.38–0.54)	0.407	0.007
**Race/ethnicity** ¶								
Mexican American	1,005	0.66	(0.57–0.75)	765	0.72	(0.59–0.85)	0.406	0.011
Black, non-Hispanic	1,145	0.55	(0.47–0.62)	566	0.62	(0.53–0.71)	0.170	0.012
White, non-Hispanic	930	0.52	(0.44–0.59)	988	0.59	(0.50–0.67)	0.108	0.015
**Income to poverty ratio**								
<130%	1,505	0.56	(0.47–0.65)	1,332	0.59	(0.53–0.66)	0.485	0.006
130% to <349%	1,196	0.49	(0.43–0.55)	949	0.63	(0.52–0.73)	0.013	0.025
≥349%	647	0.65	(0.57–0.74)	549	0.65	(0.58–0.72)	0.252	0.011
**Obesity status**								
Obese**	625	0.45	(0.36–0.53)	523	0.61	(0.54–0.67)	0.002	0.030
Not obese	2,723	0.57	(0.51–0.64)	2,307	0.62	(0.56–0.68)	0.094	0.012

**Abbreviation:** CI = confidence interval.

* Estimates age adjusted to the 2000 U.S. population.

† p-value for average annual change from 2003 to 2010 estimated using linear regression.

§ Average annual change from 2003 to 2010 in cup-equivalents per 1,000 calories calculated using linear regression.

¶ Other racial/ethnic groups not shown; all racial/ethnic groups included in reported values for the total population and values shown by sex, age groups, family income to poverty ratio, and obesity status.

** Body mass index ≥95th percentile using 2000 CDC growth chart.

**TABLE 2:** Mean intake of total vegetables in cup-equivalents per 1,000 calories among children aged 2–18 years, by sex, age, race/ethnicity, family income to poverty ratio, and obesity status — National Health and Nutrition Examination Survey, United States, 2003 to 2010*

	2003–2004			2009–2010				
				
Characteristic	No. surveyed	Mean intake	(95% CI)	No. surveyed	Mean intake	(95% CI)	p-value†	Annual change§
**All children**	**3,348**	**0.54**	**(0.50–0.57)**	**2,830**	**0.53**	**(0.49–0.58)**	**0.650**	**−0.002**
**Sex**								
Male	1,663	0.52	(0.48–0.55)	1,475	0.48	(0.45–0.51)	0.160	−0.005
Female	1,685	0.56	(0.51–0.60)	1,355	0.58	(0.50–0.66)	0.865	0.001
**Age group (yrs)**								
2–5	706	0.49	(0.45–0.54)	774	0.48	(0.44–0.52)	0.524	−0.003
6–11	861	0.55	(0.49–0.61)	1,058	0.48	(0.42–0.54)	0.080	−0.011
12–18	1,781	0.55	(0.51–0.58)	998	0.60	(0.51–0.68)	0.279	0.007
**Race/ethnicity** ¶								
Mexican American	1,005	0.61	(0.57–0.65)	765	0.56	(0.52–0.60)	0.041	−0.008
Black, non-Hispanic	1,145	0.54	(0.51–0.57)	566	0.48	(0.43–0.53)	0.047	−0.009
White, non-Hispanic	930	0.51	(0.47–0.56)	988	0.54	(0.47–0.61)	0.572	0.003
**Income to poverty ratio**								
<130%	1,505	0.56	(0.53–0.60)	1,332	0.53	(0.48–0.58)	0.460	−0.004
130% to <349%	1,196	0.56	(0.51–0.61)	949	0.53	(0.42–0.64)	0.400	−0.007
≥349%	647	0.47	(0.41–0.52)	549	0.53	(0.47–0.59)	0.300	0.006
**Obesity status**								
Obese**	625	0.56	(0.49–0.63)	523	0.52	(0.48–0.57)	0.316	−0.006
Not obese	2,723	0.53	(0.49–0.57)	2,307	0.54	(0.48–0.59)	0.828	−0.001

**Abbreviation:** CI= confidence interval.

* Estimates age adjusted to the 2000 U.S. population.

† p-value for average annual change from 2003 to 2010 estimated using linear regression.

§ Average annual change from 2003 to 2010 in cup-equivalents per 1,000 calories calculated using linear regression.

¶ Other racial/ethnic groups not shown; all racial/ethnic groups included in reported values for the total population and values shown by sex, age groups, family income to poverty ratio, and obesity status.

** Body mass index ≥ 95th percentile using 2000 CDC growth chart.

## Footnotes

* Additional information available at http://www.healthypeople.gov/2020/topicsobjectives2020/objectiveslist.aspx?topicId=29.

† Additional information available at http://myplate.gov/printpages/myplatefoodgroups/fruits/food-groups.fruits-counts.pdf and http://myplate.gov/printpages/myplatefoodgroups/vegetables/food-groups.vegetables-counts.pdf.

§ Additional information available at http://www.cdc.gov/nchs/nhanes.htm.

¶ Additional information available at http://www.ars.usda.gov/Services/docs.htm?docid=7710.

** Additional information available at http://www.ars.usda.gov/services/docs.htm?docid=23868 and http://www.cnpp.usda.gov/publications/mped/cnpp-mpedaddendumdocumentation.pdf.

†† Available at http://www.cdc.gov/nchs/data/series/sr_11/sr11_246.pdf.

§§ Additional information available at http://aspe.hhs.gov/poverty/13poverty.cfm.

¶¶ Child Nutrition and WIC Reauthorization Act of 2004. 118 Stat. 729, Sect. 101–502, 108th Congress, 2004.

*** Additional information available at http://www.fns.usda.gov/cnd/governance/legislation/cnr_2010.htm.

††† Additional information available at http://www.ecfr.gov/cgi-bin/text-idx?SID=6e619efd3476fc185e85495e42f62127&node=7:4.1.1.1.1.3.1.2&rgn=div8 for the National School Lunch Program and http://www.ecfr.gov/cgi-bin/text-idx?rgn=div5&node=7:4.1.1.1.3#7:4.1.1.1.3.0.1.8 for the National School Breakfast Program.

§§§ Richard B. Russell National School Lunch Act. P.L. 113-75, Sect. 9 [1758] Nutritional and other program requirements; Sect. 17 [1766] Child and adult care food program.

¶¶¶ Additional information available at http://www.cdc.gov/chronicdisease/about/state-public-health-actions.htm.

**** Additional information available at http://www.letsmove.gov.

## References

[1] US Department of Agriculture; US Department of Health and Human Services (2012). Dietary guidelines for Americans, 2010.

[2] National Cancer Institute Usual dietary intakes: food intakes, US population, 2007–10.

[3] Due P, Krolner R, Rasmussen M (2011). Pathways and mechanisms in adolescence contribute to adult health inequalities. Scand J Public Health.

[4] Hazel ABH, Guenther PM, Rihane CI (2013). Diet quality of children age 2–17 years as measured by the healthy eating index—2010.

[5] American Academy of Pediatrics Committee on Nutrition (2001). The use and misuse of fruit juice in pediatrics. Pediatrics.

[6] Blanchette L, Brug J (2005). Determinants of fruit and vegetable consumption among 6–12-year-old children and effective interventions to increase consumption. J Hum Nutr Diet.

[7] US Department of Agriculture (2007). Special Supplemental Nutrition Program for Women, Infants and Children (WIC): revisions in the WIC food packages—interim rule.

[8] Whaley SE, Ritchie LD, Spector P (2012). Revised WIC food package improves diets of WIC families. J Nutr Educ Behav.

[9] US Department of Agriculture (2013). Evaluation of the Fresh Fruit And Vegetable Program—summary 2013.

[10] CDC (2012). Competitive foods and beverages in US schools: a state policy analysis.

[11] CDC (2009). State indicator report on fruits and vegetables, 2009.

[12] CDC (2011). Weight of the Nation early care and education policy review.

[13] Institute of Medicine (2012). Accelerating progress in obesity prevention: solving the weight of the nation.

[14] US Department of Health and Human Services (2010). The Surgeon General’s vision for a healthy and fit nation.

[15] US Department of Commerce, US Census Bureau (2013). Who’s minding the kids? Child care arrangements: spring 2011. Report no. P70-135.

[16] National Center for Education Statistics (2012). Digest of education statistics. Table 105.30. Enrollment in educational institutions, by level and control of institution: selected years, 1869–70 through fall 2023.

[17] Cullen KW, Watson K, Zakeri I (2008). Improvements in middle school student dietary intake after implementation of the Texas public school nutrition policy. Am J Public Health.

[18] Newman C (2013). Fruit and vegetable consumption by school lunch participants implications for the success of new nutrition standards.

[19] CDC (2011). School health guidelines to promote healthy eating and physical activity. MMWR.

[20] Institute of Medicine (2011). Early childhood obesity prevention policies.

[21] Institute of Medicine (2011). Child and Adult Care Food Program: aligning dietary guidance for all.

[22] American Academy of Pediatrics; American Public Health Association; National Resource Center for Health and Safety in Child Care and Early Education (2012). Preventing childhood obesity in early care and education.

[23] Thompson FE, Byers T (1994). Dietary assessment resource manual. J Nutr.

[24] Livingstone MBE, Robson PJ, Wallace JMW (2004). Issues in dietary intake assessment of children and adolescents. Brit J Nutr.

